# Primary pulmonary choriocarcinoma with brain metastases in a male: a case report and literature review

**DOI:** 10.3389/fonc.2026.1945521

**Published:** 2026-10-20

**Authors:** Zhipeng Yin, Ying Xiao, Dong Zhang, Bo Liu, Shufang Liu

**Affiliations:** 1 Department of Clinical Laboratory, Linyi Central Hospital, Linyi, Shandong, China; 2 Department of Gastroenterology, Linyi Central Hospital, Linyi, Shandong, China; 3 Department of Respiratory and Critical Care Medicine, Shandong Medical and Pharmaceutical University Hospital, Shandong Medical and Pharmaceutical University, Binzhou, Shandong, China

**Keywords:** brain metastasis, chemotherapy, human chorionic gonadotropin, immunotherapy, male, primary pulmonary choriocarcinoma

## Abstract

**Background:**

Primary pulmonary choriocarcinoma (PPC) is an exceptionally rare and highly aggressive extragonadal trophoblastic malignancy in males, with no established standard treatment. Cytotoxic chemotherapy alone generally yields limited therapeutic benefit, while the role of immune checkpoint inhibitors (ICIs) remains poorly defined.

**Case presentation:**

Here, we report a 41-year-old man who presented with a six-month history of cough and was found to have a large right upper lobe pulmonary mass with multiple brain metastases. Histopathological examination revealed typical trophoblastic differentiation, and immunohistochemistry demonstrated diffuse positivity for HCG, CK7, GATA3, and SALL4, whereas TTF-1 and Napsin A were negative. Serum β-human chorionic gonadotropin (β-HCG) was markedly elevated (> 10, 000 mIU/mL), and no gonadal primary lesion was identified, confirming the diagnosis of primary pulmonary choriocarcinoma.

**Intervention/outcome:**

The patient underwent multimodal treatment including radiotherapy, EMA/CO chemotherapy, EMA/EP combined with sintilimab, and third-line therapy consisting of nab-paclitaxel, bevacizumab, and penpulimab. Despite intensive treatment, the disease progressed rapidly with new intracranial and systemic metastases. Throughout the disease course, serial β-HCG measurements closely paralleled radiographic findings and provided timely guidance for therapeutic decision-making. In addition, we reviewed previously reported metastatic male PPC cases and performed an exploratory survival analysis, which suggested that patients receiving ICI-based therapy tended to have longer overall survival than those treated without immunotherapy.

**Conclusion:**

Although these findings should be interpreted cautiously because of the limited sample size, this case highlights the highly aggressive nature of male PPC, emphasizes the diagnostic and monitoring value of dynamic β-HCG assessment, and supports further investigation of chemoimmunotherapy as a potential treatment strategy for this rare malignancy.

## 1 Introduction

Choriocarcinoma is a malignant trophoblastic neoplasm characterized by the production of human chorionic gonadotropin (HCG) (1). Most cases are gestational in origin and arise from the uterine chorionic villi following pregnancy (2). In contrast, primary pulmonary choriocarcinoma (PPC) is a non-gestational extragonadal trophoblastic tumor that occurs predominantly in the lung and is exceedingly rare, particularly in males. Because of its rarity, the biological behavior, optimal management, and prognostic factors of male PPC remain poorly understood (3).

PPC is characterized by rapid tumor growth, extensive hemorrhage and necrosis, early hematogenous dissemination, and a dismal prognosis (3). Clinical manifestations are nonspecific and commonly include cough, hemoptysis, chest pain, and dyspnea, often leading to initial misdiagnosis as conventional lung carcinoma (4). Histopathological examination combined with immunohistochemistry remains the diagnostic cornerstone, with diffuse HCG expression in syncytiotrophoblastic cells serving as the most important diagnostic feature (7, 10).

Currently, no standardized treatment guidelines exist for PPC (3). Therapeutic strategies are generally extrapolated from gestational trophoblastic neoplasia and non-seminomatous germ cell tumors, including EMA/CO, EMA/EP, PEB, and BEP regimens (13–16). However, treatment responses are frequently unsatisfactory, and chemoresistance develops rapidly in advanced disease (3, 4). Recent studies have demonstrated widespread PD-L1 expression in trophoblastic tumors, providing a biological rationale for the use of immune checkpoint inhibitors (ICIs) (5). Several case reports have described encouraging responses to PD-1/PD-L1 blockade, but clinical experience in male PPC remains limited (8, 9, 11, 12).

Herein, we report a 41-year-old male with stage IV primary pulmonary choriocarcinoma accompanied by multiple brain metastases. The patient underwent sequential multimodal treatment, including chemotherapy, radiotherapy, anti-angiogenic therapy, and immunotherapy, while serum β-HCG was monitored dynamically throughout the disease course. In addition, we performed a systematic review of previously reported male PPC cases and conducted exploratory survival analyses to evaluate the potential prognostic significance of baseline β-HCG and the association between ICI-based therapy and overall survival. This report aims to enhance awareness of this rare malignancy and provide additional evidence regarding biomarker-guided management and emerging immunotherapeutic strategies.

## 2 Case report

A 41-year-old man presented to our hospital with a six-month history of intermittent cough. He had a 20-year smoking history of 30 cigarettes per day and had ceased smoking. There was no history of chronic respiratory disease, occupational exposure to toxic dust, or prior malignancy. Physical examination findings were unremarkable; no palpable testicular mass or gynecomastia was detected.

Regarding past medical, personal, and family history, the patient was generally well, without a history of hepatitis, tuberculosis, hypertension, diabetes mellitus, cardiovascular disease, or psychiatric disorders. No surgical, traumatic, or blood transfusion history was documented, and no known drug hypersensitivity was identified. The patient was a long-term resident of Shandong Province, with no documented exposure to epidemic agents or radioactive materials. He married at 25 years of age; both his spouse and one daughter were in good health. No familial or genetic tumor predisposition was identified, and no family members had a history of comparable malignant disease.

Initial imaging examinations revealed extensive pulmonary and intracranial metastatic disease. Chest computed tomography (CT) demonstrated a large irregular mass measuring approximately 10.2 × 8.6 cm in the upper lobe of the right lung, with heterogeneous density and mild heterogeneous enhancement after contrast administration. Multiple small nodules were identified in the left lung, suggesting intrapulmonary metastatic lesions (**Figures 1A, B**). Brain magnetic resonance imaging (MRI) showed multiple intracranial lesions accompanied by surrounding edema. Subsequent contrast-enhanced brain magnetic resonance imaging (MRI) confirmed multiple metastatic lesions involving the right cerebellar hemisphere and cerebellar vermis, with the largest lesion measuring approximately 3.6 × 3.3 cm, demonstrating heterogeneous enhancement and features consistent with brain metastases (**Figures 1C, D**).

**Figure 1:**
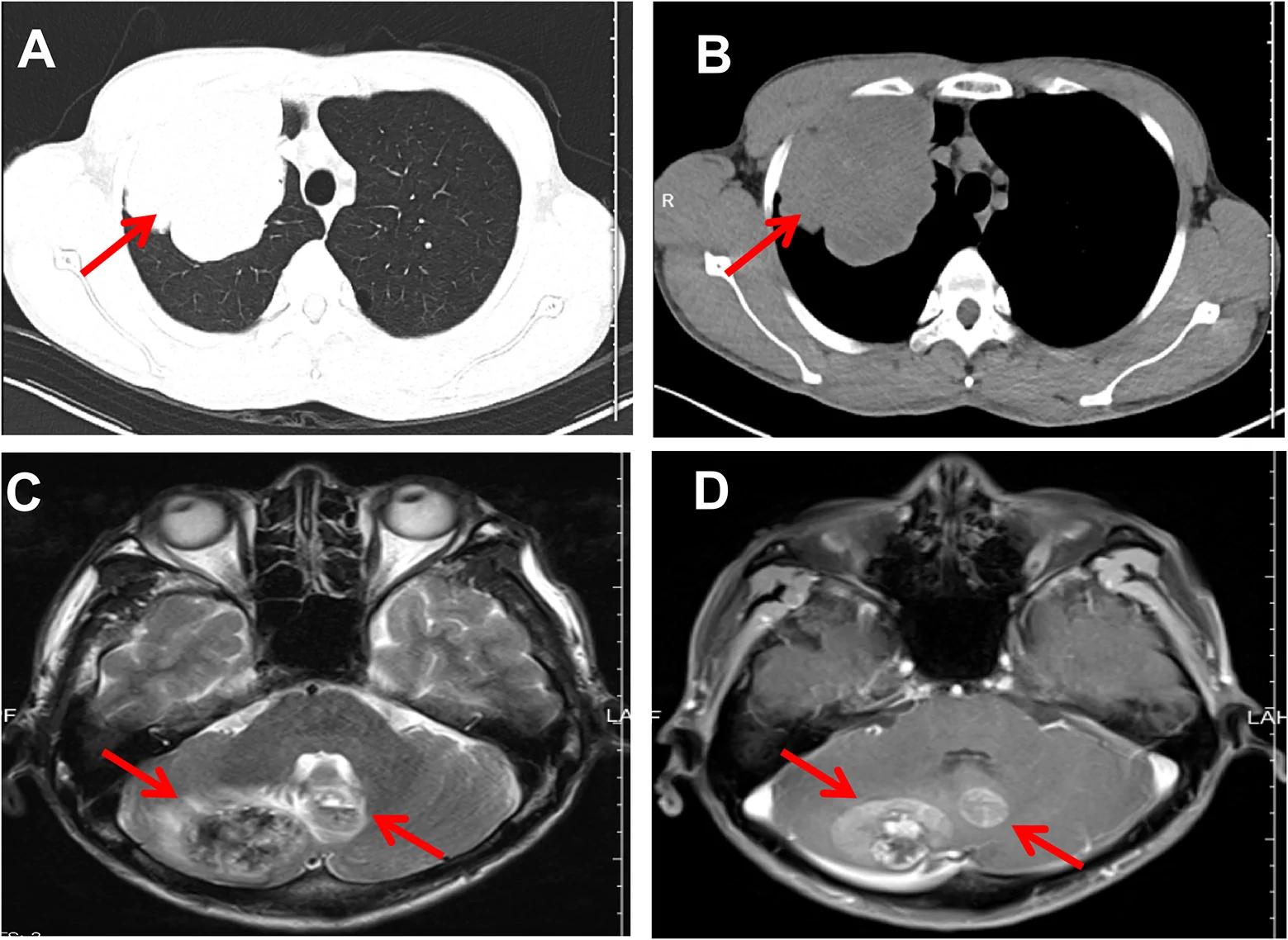
Initial radiological findings of primary pulmonary choriocarcinoma with brain metastases. **(A)** Chest CT lung window showing a large irregular mass in the upper lobe of the right lung with multiple pulmonary nodules. **(B)** Chest CT mediastinal window demonstrating the extent of the primary pulmonary lesion. **(C)** Brain CT plain scan showing multiple intracranial lesions with surrounding edema. **(D)** Contrast-enhanced brain MRI showing multiple metastatic lesions with heterogeneous enhancement.

Routine laboratory investigations were largely unremarkable except for a markedly elevated serum β-human chorionic gonadotropin (β-HCG) level exceeding 10, 000 mIU/mL.

CT-guided percutaneous biopsy of the pulmonary mass demonstrated extensive hemorrhage and necrosis with malignant trophoblastic cells. Histologically, the tumor consisted of cytotrophoblasts admixed with multinucleated syncytiotrophoblasts, consistent with biphasic trophoblastic differentiation. Hematoxylin and eosin (H&E) staining at different magnifications demonstrated the characteristic biphasic proliferation pattern of mononuclear cytotrophoblasts and multinucleated syncytiotrophoblasts, which are typical pathological features of choriocarcinoma (**Figures 2A, B**). Immunohistochemical analysis showed diffuse positivity for β-HCG (3+), CK7 (3+), GATA3 (3+), and SALL4 (2+) (**Figures 2C–F**), supporting trophoblastic differentiation. Focal positivity for p40 and a high Ki-67 labeling index (~70%) were observed, whereas TTF-1, Napsin A, CK5/6, and Glypican-3 were negative (**Supplementary Figure 1**).

**Figure 2:**
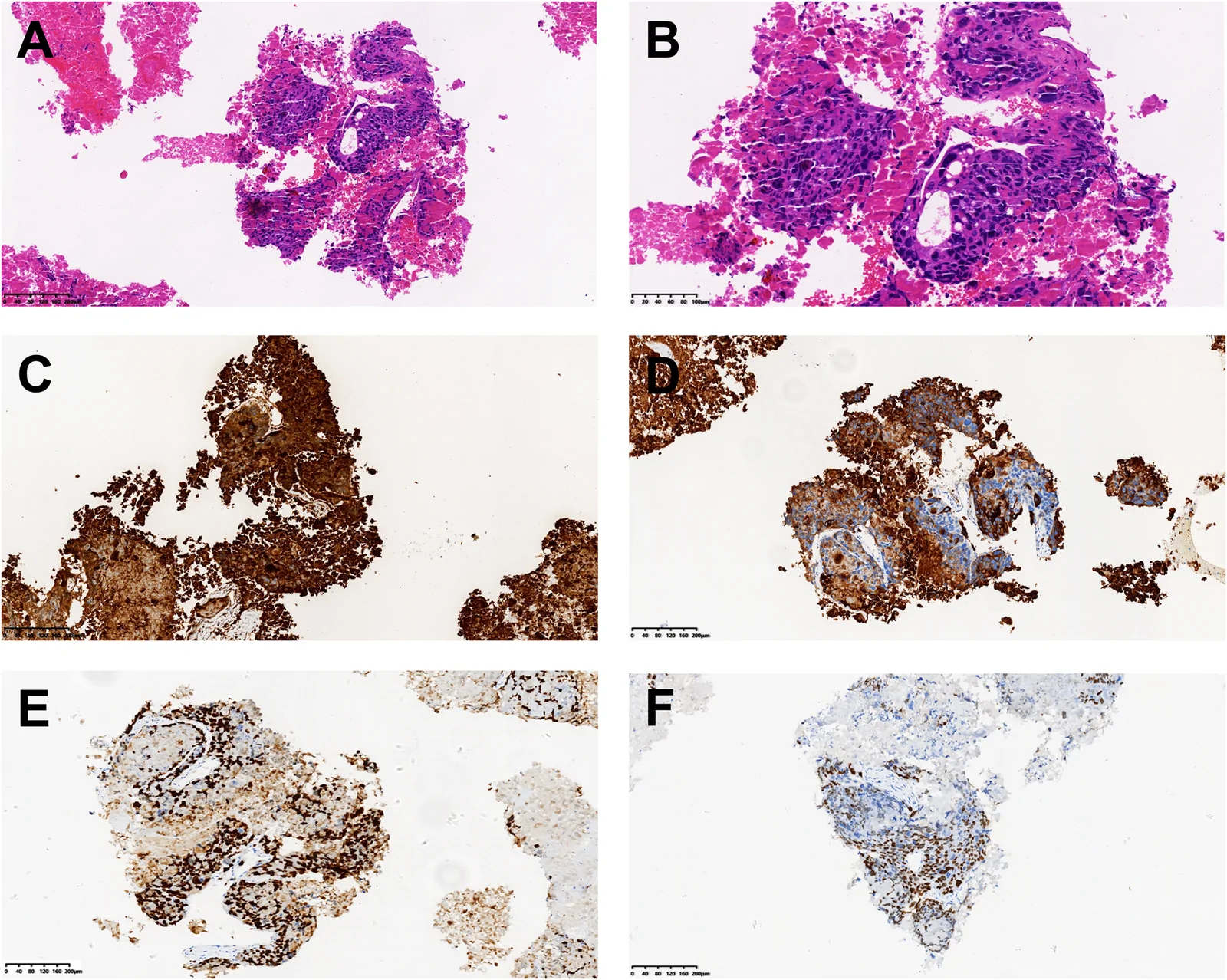
Histopathological and immunohistochemical features of primary pulmonary choriocarcinoma. **(A)** Hematoxylin-eosin (H&E) staining, scale bar = 200 μm. Low-magnification view of fragmented lung biopsy tissue showing extensive hemorrhage, necrosis, and multiple nests of viable tumor cells. **(B)** Hematoxylin-eosin (H&E) staining, scale bar = 100 μm. High-magnification view demonstrating biphasic proliferation of mononuclear cytotrophoblasts and multinucleated syncytiotrophoblasts with prominent nuclear atypia, hemorrhage, and necrosis. **(C)** Immunohistochemical staining for β-HCG (3+), scale bar = 200 μm. Strong diffuse cytoplasmic immunoreactivity predominantly in syncytiotrophoblastic cells. **(D)** Immunohistochemical staining for CK7 (3+), scale bar = 200 μm. Robust positivity within tumor cells. **(E)** Immunohistochemical staining for GATA3 (3+), scale bar = 200 μm. Diffuse nuclear positivity in tumor cells. **(F)** Immunohistochemical staining for SALL4 (2+), scale bar = 200 μm. Positive nuclear staining of tumor cells.

Because focal p40 expression may mimic poorly differentiated squamous cell carcinoma, careful pathological interpretation was required. The combination of characteristic trophoblastic morphology, a specific immunophenotypic profile, markedly elevated serum β-HCG levels, and the absence of palpable testicular abnormalities on physical examination supported the diagnosis of primary pulmonary choriocarcinoma (PPC). No clinically evident alternative primary tumor was identified during the initial evaluation. However, exclusion of a gonadal primary tumor was based on physical examination alone, as scrotal ultrasonography was not performed. Therefore, an occult or regressed testicular primary choriocarcinoma could not be completely excluded. In addition, genomic profiling by next-generation sequencing (NGS) and biomarker testing for programmed death-ligand 1 (PD-L1) expression, tumor mutational burden (TMB), and microsatellite instability (MSI) were not performed because of financial constraints. Nevertheless, the predominant pulmonary mass, characteristic biphasic trophoblastic morphology, immunohistochemical profile, and markedly elevated serum β-HCG level supported a diagnosis most consistent with primary pulmonary choriocarcinoma. Given the multiple intracranial metastatic lesions, the disease was considered advanced PPC with brain metastases.

The patient initially received radiotherapy for brain metastases on June 16, 2025; however, treatment was discontinued because of poor tolerance. Subsequently, systemic chemotherapy with the EMA/CO regimen was initiated on June 18, 2025. The patient received three cycles of EMA/CO chemotherapy, starting on June 18, July 14, and August 7, 2025, respectively. During the early phase of treatment, serum β-HCG levels showed a transient decline from the initial extremely elevated level; however, this response was not sustained. Follow-up evaluation demonstrated progressive elevation of serum β-HCG accompanied by radiological progression, including the development of new metastatic lesions in the left cerebellar hemisphere, right frontal lobe, and left thalamus (**Figures 3A, C**).

**Figure 3:**
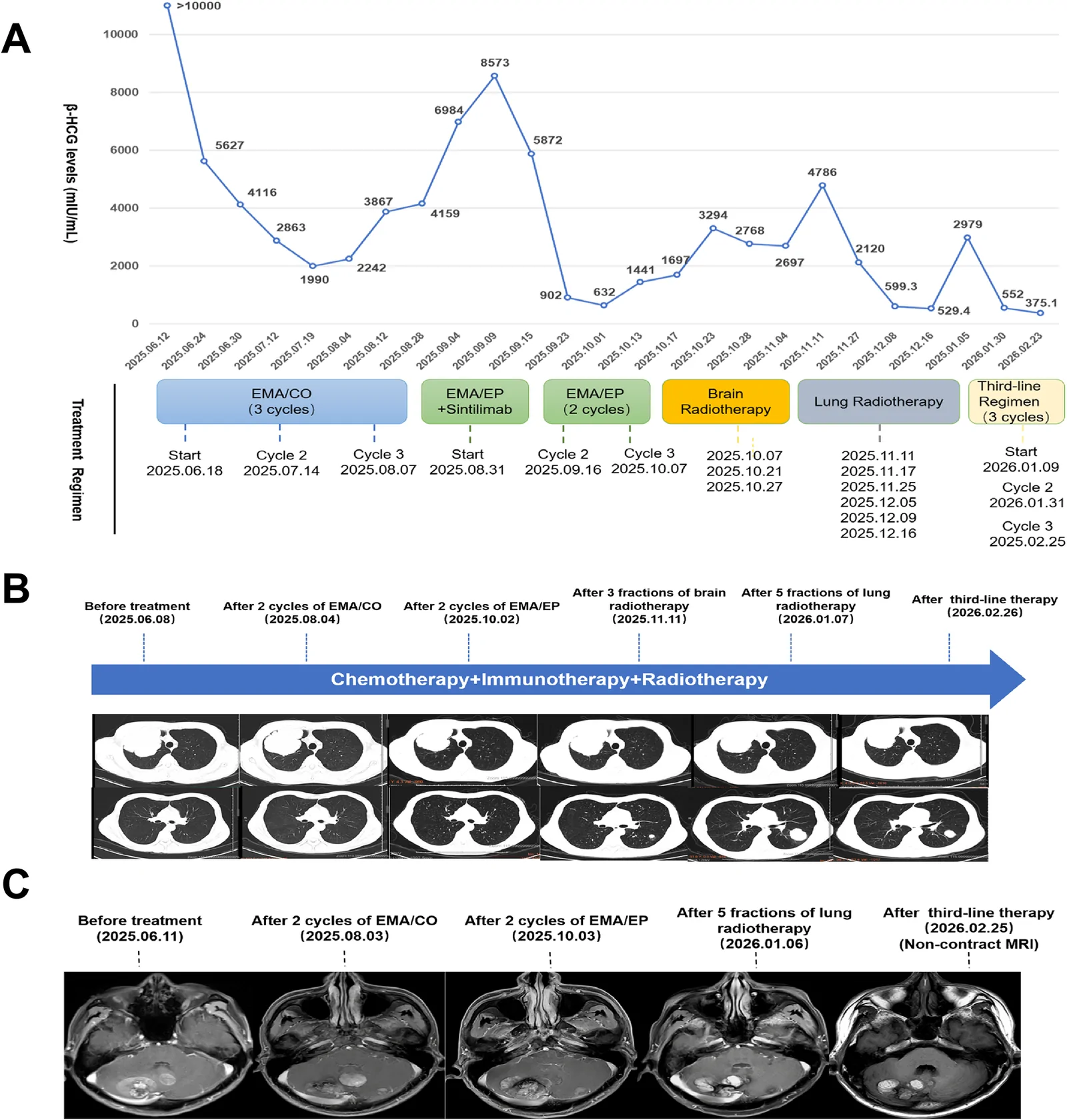
Dynamic changes in serum β-HCG levels, imaging findings, and treatment course during disease progression. **(A)** Serial serum β-HCG levels and corresponding treatments, including EMA/CO, EMA/EP with or without sintilimab, brain radiotherapy, thoracic radiotherapy, and third-line therapy with nab-paclitaxel, bevacizumab, and penpulimab. **(B)**Serial chest CT images showing changes in the primary pulmonary lesion and metastatic pulmonary nodules before and after treatment. Images are arranged chronologically from left to right, with the examination dates and treatment status indicated above each image set. **(C)** Serial brain MRI images demonstrating the temporal evolution of intracranial metastatic lesions. Representative images are arranged chronologically from left to right, beginning with the pretreatment baseline examination and followed by examinations performed after two cycles of EMA/CO chemotherapy, after two cycles of EMA/EP chemotherapy, after five fractions of thoracic radiotherapy, and after third-line therapy. The corresponding examination dates and treatment status are indicated above each image set. The MRI obtained after third-line therapy was a non-contrast examination.

Given disease progression after EMA/CO chemotherapy, the treatment strategy was modified to the EMA/EP regimen on August 31, 2025. Sintilimab, an anti-programmed death-1 (PD-1) immune checkpoint inhibitor, was administered during the first EMA/EP cycle. Subsequent EMA/EP cycles were continued without sintilimab, with treatment dates on September 16 and October 7, 2025. Although serum β-HCG levels initially decreased after switching chemotherapy regimens, the patient continued to exhibit disease progression, characterized by persistent pulmonary lesions and progressive intracranial metastases on serial imaging (**Figures 3A–C**).

During this period, brain radiotherapy was performed because of progressive intracranial metastatic disease, beginning on October 7, 2025. Serial brain MRI demonstrated persistent and progressive metastatic lesions despite local treatment (**Figure 3C**). In addition, thoracic radiotherapy was subsequently administered from November 11, 2025, because of progressive pulmonary lesions. Serial chest CT examinations showed continued changes in the primary pulmonary lesion and metastatic nodules during treatment (**Figure 3B**).

Because of further systemic progression despite multiple lines of chemotherapy, immunotherapy, and radiotherapy, a third-line treatment regimen consisting of nab-paclitaxel-based therapy combined with bevacizumab and penpulimab was initiated on January 9, 2026, followed by subsequent cycles on January 31 and February 25, 2026. Although serum β-HCG levels temporarily decreased after third-line treatment, the patient eventually developed rapid disease progression with persistent pulmonary and intracranial metastatic lesions (**Figures 3A–C**). Active anticancer treatment was ultimately discontinued after discussion with the patient’s family.

Following discontinuation of all active anticancer therapies, the patient and his family declined further oncological interventions after hospital discharge, and only best supportive and palliative symptomatic care was administered. The patient was discharged on March 4, 2026. The total follow-up duration from pathological diagnosis (June 13, 2025) to the last follow-up (March 4, 2026) was approximately 8.6 months. At the time of manuscript preparation, the patient remained alive with poor performance status secondary to persistent progressive intracranial and systemic tumor burden.

From the clinician’s perspective, after hospital discharge on March 4, 2026, the patient received home-based best-supportive palliative care and no further hospital-based laboratory or imaging follow-up examinations were performed. Therefore, post-discharge objective laboratory and imaging data are unavailable.In terms of treatment adherence and tolerance during inpatient multimodal therapy, the patient completed scheduled cycles of chemotherapy, immunotherapy and radiotherapy as planned; treatment interruption was only caused by poor tolerance to initial brain radiotherapy. No unexpected adverse events or treatment-related complications were observed throughout the inpatient treatment period.

From the patient’s perspective, the patient reported substantial psychological distress and anxiety regarding the malignant prognosis during hospitalization. Despite the heavy disease burden, the patient understood the disease condition and the goals of palliative care. Written informed consent was obtained for reporting these subjective experiences in this case report.

Throughout the disease course, dynamic serum β-HCG monitoring closely reflected tumor progression and treatment response. Initial reductions in β-HCG levels were associated with temporary therapeutic responses, whereas subsequent increases corresponded with radiographic progression, suggesting that serial β-HCG measurement may serve as a valuable biomarker for monitoring disease burden and therapeutic efficacy in patients with primary pulmonary choriocarcinoma (**Figure 3A**).

Serial serum β-HCG measurements were obtained throughout the disease course and closely paralleled radiographic findings. Changes in β-HCG preceded or accompanied alterations in tumor burden, allowing timely assessment of treatment response and early recognition of therapeutic resistance. The concordance between serum β-HCG kinetics and imaging findings suggests that dynamic β-HCG monitoring may represent a valuable surrogate biomarker for disease burden and treatment efficacy in patients with PPC.

## 3 Methods

A literature search was performed in PubMed using the keywords “primary pulmonary choriocarcinoma” and related terms. Eligible studies published in English were screened, and cases of male patients with confirmed PPC were included. Cases without sufficient clinical information, pathological confirmation, or survival data were excluded. The following information was extracted from each eligible report: first author, year of publication, patient age, initial clinical presentation, metastatic sites, pretreatment serum β-human chorionic gonadotropin (β-HCG) level (mIU/mL), treatment regimen, and survival data. The optimal cut-off value for pretreatment β-HCG in the Kaplan–Meier survival analysis was determined using X-tile software.

Overall survival (OS) was defined as the interval from the date of diagnosis to death from any cause or the last follow-up date. Kaplan–Meier survival curves were generated to estimate OS, and differences between groups were evaluated using the log-rank test. Exploratory Cox proportional hazards regression analysis was performed to investigate potential prognostic factors. Statistical analyses were conducted using GraphPad Prism software, and a two-sided P value <0.05 was considered statistically significant.

Given the rarity of PPC and the limited number of reported cases, all survival analyses were considered exploratory and hypothesis-generating rather than confirmatory.

## 4 Literature review and discussion

Primary pulmonary choriocarcinoma (PPC) is an exceptionally rare and highly aggressive extragonadal trophoblastic malignancy that predominantly affects young and middle-aged men. Owing to its rarity, the biological characteristics, optimal treatment strategies, and prognostic factors remain poorly understood (3, 4). Compared with gestational choriocarcinoma, PPC exhibits a more aggressive clinical course, with extensive hemorrhage and necrosis, rapid hematogenous dissemination, and poor responsiveness to conventional chemotherapy, resulting in an unfavorable prognosis (17). Because published evidence regarding prognostic factors and the efficacy of immune checkpoint inhibitor (ICI)-based therapy in male PPC remains extremely limited, we systematically reviewed all eligible published cases and performed exploratory Kaplan–Meier survival analyses to evaluate the associations between baseline serum β-HCG, ICI-based therapy, and overall survival. In our literature review, we identified 33 metastatic male PPC cases with available survival information and an additional 18 male PPC cases with pretreatment serum β-human chorionic gonadotropin (β-HCG) data suitable for prognostic analysis (**Supplementary Tables 1**, **2**). These two datasets were analyzed independently because they were derived from different published reports and should not be regarded as nested cohorts. Kaplan–Meier survival analysis demonstrated that patients receiving immune checkpoint inhibitor (ICI)-based therapy achieved significantly longer overall survival than those treated without immunotherapy (**Figure 4A**). Likewise, patients with lower pretreatment serum β-HCG levels had significantly better overall survival than those with markedly elevated β-HCG levels (**Figure 4B**). These exploratory findings provide preliminary evidence supporting the prognostic value of baseline β-HCG and the potential therapeutic role of immunotherapy; however, these observations should be interpreted cautiously because of the limited sample size, heterogeneous treatment strategies, and inevitable publication bias.

**Figure 4:**
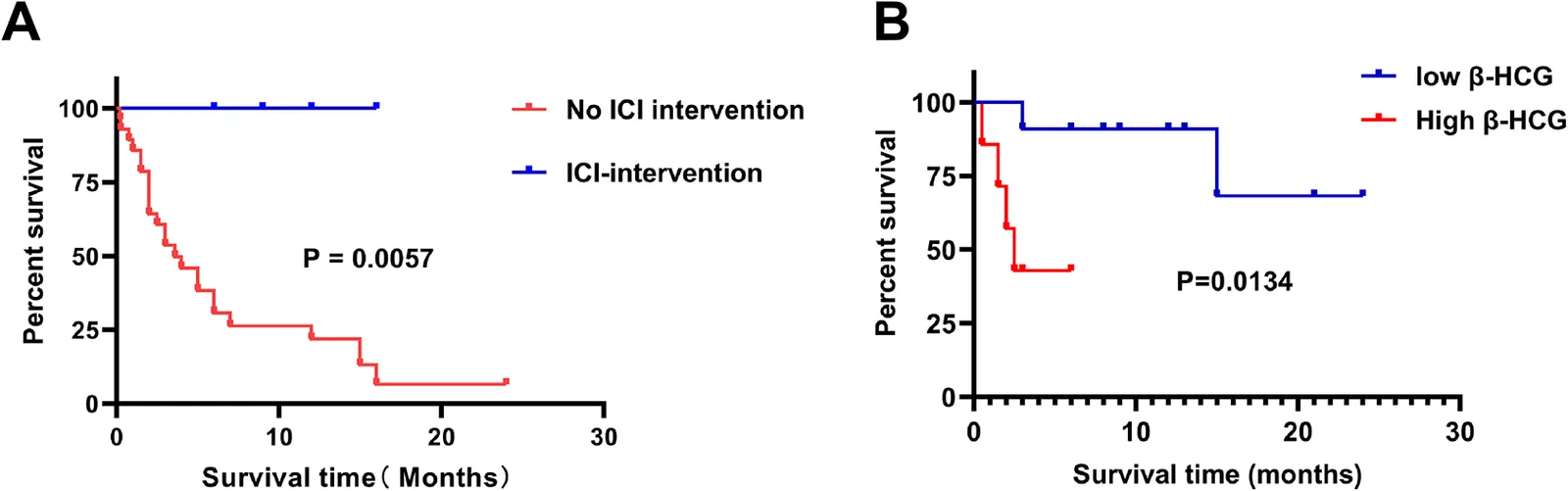
Kaplan–Meier survival analyses of prognostic factors in male patients with primary pulmonary choriocarcinoma. **(A)** Overall survival according to immune checkpoint inhibitor (ICI)-based therapy (log-rank P = 0.0057). **(B)** Overall survival according to pretreatment serum β-HCG levels (log-rank P = 0.0134).

The diagnosis of PPC remains particularly challenging because its clinical manifestations are nonspecific and its pathological features may overlap with those of poorly differentiated lung carcinoma (3). Most patients initially present with cough, hemoptysis, chest pain, or dyspnea (4), whereas endocrine manifestations related to excessive HCG secretion, including gynecomastia or reduced libido, are relatively uncommon and may easily be overlooked (6). Consequently, many patients are diagnosed only after developing advanced metastatic disease. Histologically, PPC is characterized by a biphasic proliferation of cytotrophoblasts and multinucleated syncytiotrophoblasts accompanied by extensive hemorrhage and necrosis (7). However, these characteristic features may be difficult to appreciate in small biopsy specimens, increasing the risk of misdiagnosis. In the present case, focal p40 expression initially raised the possibility of poorly differentiated squamous cell carcinoma, representing an important diagnostic pitfall. Previous studies have also reported focal expression of squamous markers, including p40, p63, and CK5/6, in some PPC cases, indicating that diagnosis should never rely on a single immunohistochemical marker. Instead, a comprehensive evaluation integrating tumor morphology, immunophenotypic findings, serum β-HCG levels, and careful exclusion of a gonadal primary lesion is essential. In our patient, the characteristic trophoblastic morphology, diffuse HCG expression, positivity for GATA3 and SALL4, markedly elevated serum β-HCG, and the absence of a testicular primary tumor collectively established the diagnosis of PPC. Therefore, serum β-HCG measurement should be considered routinely in male patients presenting with rapidly progressive pulmonary masses showing unusual pathological or immunohistochemical features.

Several diagnostic limitations of the present case should be acknowledged. Although the predominant pulmonary mass, characteristic biphasic trophoblastic morphology, diffuse β-HCG expression, supportive immunohistochemical profile, and markedly elevated serum β-HCG level favored primary pulmonary choriocarcinoma, a gonadal primary tumor was excluded only by physical examination. Because scrotal ultrasonography and postmortem examination were not performed, an occult or regressed testicular primary choriocarcinoma could not be definitively excluded (18). In addition, focal p40 positivity represented a potential diagnostic pitfall because it may mimic poorly differentiated pulmonary squamous cell carcinoma. Therefore, the diagnosis should be interpreted as being most consistent with PPC on the basis of the available clinicopathological evidence.

Genomic profiling by next-generation sequencing (NGS) and biomarker testing for programmed death-ligand 1 (PD-L1) expression, tumor mutational burden (TMB), and microsatellite instability (MSI) were not performed because of financial constraints. Consequently, potentially actionable genomic alterations and biomarkers associated with response to immune checkpoint inhibitors could not be evaluated. PD-L1 expression in trophoblastic tumors provides a biological rationale for PD-1/PD-L1 blockade, whereas high TMB and MSI-high status may indicate increased tumor immunogenicity and potential sensitivity to immune checkpoint inhibition in selected malignancies (19). However, the predictive value of these biomarkers has not been established in PPC. Comprehensive molecular profiling in future cases may improve diagnostic confidence, identify potential therapeutic targets, and facilitate individualized selection of immunotherapy.

Currently, no standardized treatment guidelines have been established for PPC because of the extreme rarity of the disease (3, 4). Therapeutic strategies are generally extrapolated from gestational trophoblastic neoplasia and non-seminomatous germ cell tumors, with EMA/CO, EMA/EP, BEP, and platinum-based regimens being the most commonly employed approaches (13, 16). Nevertheless, unlike gestational choriocarcinoma, which is highly sensitive to chemotherapy, PPC frequently demonstrates primary or acquired chemoresistance (14). Our patient experienced rapid disease progression despite sequential treatment with EMA/CO, EMA/EP combined with sintilimab, radiotherapy, and third-line therapy consisting of nab-paclitaxel, bevacizumab, and penpulimab, illustrating the aggressive biological behavior of advanced PPC with brain metastases. Increasing evidence suggests that trophoblastic tumors frequently express PD-L1, providing a biological rationale for immune checkpoint blockade (5). Several recent case reports have described durable responses to PD-1 or PD-L1 inhibitors in patients with chemotherapy-refractory PPC (20, 21). Consistent with these observations, our exploratory survival analysis demonstrated a trend toward prolonged overall survival among patients treated with ICI-based regimens. However, because the currently available evidence consists almost exclusively of isolated case reports with substantial heterogeneity in treatment regimens and follow-up duration, these encouraging findings should be interpreted with caution. Prospective multicenter studies incorporating comprehensive molecular profiling are warranted to identify biomarkers predictive of immunotherapy response and to optimize treatment selection for this rare malignancy.

An additional strength of the present case is the continuous monitoring of serum β-HCG throughout the disease course. Although β-HCG has traditionally been regarded as a diagnostic marker for trophoblastic tumors, our findings suggest that its clinical value extends beyond diagnosis alone. Serial β-HCG measurements closely paralleled radiographic tumor progression and therapeutic response, with changes in β-HCG occurring simultaneously with, or even preceding, imaging evidence of disease progression. These observations indicate that dynamic β-HCG monitoring may provide a simple, noninvasive, and readily repeatable surrogate marker for real-time assessment of tumor burden and early identification of treatment resistance. When interpreted together with the prognostic findings from our pooled analysis, β-HCG appears to possess both prognostic and monitoring value in male PPC. Therefore, integrating serial β-HCG assessment into routine clinical practice may facilitate earlier therapeutic adjustment and improve individualized disease management. Collectively, this case further highlights the highly aggressive nature of male PPC, underscores the importance of comprehensive pathological evaluation to avoid diagnostic pitfalls, and supports the potential role of biomarker-guided management and ICI-based therapy in improving outcomes for this exceptionally rare and challenging disease.

## 5 Conclusion

Primary pulmonary choriocarcinoma in males is an exceptionally rare and aggressive malignancy with limited treatment options and poor clinical outcomes. This case highlights the diagnostic challenges associated with PPC, particularly the potential overlap with poorly differentiated pulmonary carcinoma on pathological examination. A comprehensive diagnostic approach integrating tumor morphology, immunohistochemical profiling, and serum β-HCG assessment is essential for accurate diagnosis. Our findings further suggest that serial β-HCG monitoring may serve as a valuable biomarker for evaluating treatment response, disease progression, and prognosis in male PPC. Although exploratory survival analysis from previously reported cases indicated a potential association between ICI-based therapy and improved survival, the clinical benefit of immunotherapy requires confirmation in larger, multicenter studies. Overall, this report provides additional clinical evidence supporting biomarker-guided management and highlights the need for further investigation into effective therapeutic strategies for this rare and aggressive disease.

## Data Availability

The original contributions presented in the study are included in the article/**Supplementary Material**. Further inquiries can be directed to the corresponding authors.
