# Supplementary material for: Primary pulmonary choriocarcinoma with brain metastases in a male: a case report and literature review
Source: Front Oncol. 2026 Oct 20;16:1945521. doi: 10.3389/fonc.2026.1945521 (PMC13727472; doi:10.3389/fonc.2026.1945521)
Supplement: Supplementary file 1 [file Supplementaryfile1.docx]

**Supplementary Figure 1. Additional immunohistochemical findings for differential diagnosis of primary pulmonary choriocarcinoma.**

**A**

**B**

**C**

**D**

**E**

**F**

Additional immunohistochemical staining showing (A)P40(+), (B)Ki-67(70%), (C)TTF-1(-) (D)Napsin A (-), (E) CK5/6 (-) and (F) Glypican-3 (-).
