# Supplementary material for: Primary pulmonary choriocarcinoma with brain metastases in a male: a case report and literature review
Source: Front Oncol. 2026 Oct 20;16:1945521. doi: 10.3389/fonc.2026.1945521 (PMC13727472; doi:10.3389/fonc.2026.1945521)
Supplement: Supplementary file 2 [file Table1.docx]

**Supplementary Table 1. Clinical characteristics of metastatic male PPC patients**

| First author | publication year | Age (Year) | symptom(s) | Metastasis | Therapy | Survival time (months) | Status | Ref |
| --- | --- | --- | --- | --- | --- | --- | --- | --- |
| Kamata S | 2017 | 70 | Cough | Abdominal, brain | Operation | 24 | Alive | (1) |
| Patra S B | 1983 | 22 | Cough, dyspnea | Lung, brain, spleen | Chemotherapy | 0.77 | Dead | (2) |
| Ma Y | 2018 | 71 | Cough, hemoptysis | Brain | Operation | 3 | Dead | (3) |
| Hayakawa K | 1977 | 45 | Gynecomastia | Lung, liver, kidney, | radiotherapy | 5 | Dead | (4) |
| Hayakawa K | 1977 | 57 | Cough, gynecomastia | Brain | radiotherapy | 12 | Dead | (4) |
| Hattori M | 1979 | 63 | Cough, dyspnea, gynecomastia | Lung, pleura, diaphragm, pericardium, lymph nodes | Chemotherapy | 2 | Dead | (5) |
| Endou T | 1988 | 37 | Upper abdomen pain, gynecomastia | Lung, liver, brain, spleen, kidney, intestine | Chemotherapy | 2.5 | Dead | (6) |
| Adachi H, | 1989 | 71 | Dyspnea, chest pain, upper abdomen pain, gynecomastia | Lung, liver, kidney, adrenal gland, bone marrow and lymph nodes | Others | 6 | Dead | (7) |
| Sridhar KS | 1989 | 37 | Cough, chest pain, hemoptysis, fever | Lung and brain | Operation, Chemotherapy and radiotherapy | 15 | Dead | (8) |
| Sullivan LG | 1989 | 51 | Cough, weight loss | Lung, brain, liver, spleen | Radiotherapy | 0.23 | Dead | (9) |
| Durieu I | 1994 | 61 | Hemoptysis, chest pain | Lung | Operation, radiotherapy, chemotherapy | 1.5 | Dead | (10) |
| Otsuka T | 1994 | 0.33(4m) | Pubic hair, enlarged penis, pigmented scrotal skin | Brain | Operation, chemotherapy, radiotherapy, | 7 | Dead | (11) |
| Toda S | 1995 | 69 | Hemoptysis, weight loss | Lung, liver, adrenals, lymph nodes | Chemotherapy | 1.5 | Dead | (12) |
| Ikura Y | 2000 | 60 | None | Lung, brain | Chemotherapy | 5 | Dead | (13) |
| Chen F | 2001 | 61 | Hemoptysis | Lung | Operation, chemotherapy | 6 | Alive | (14) |
| Tsai JR | 2002 | 23 | Hemoptysis, dyspnea | NA | Chemotherapy | 0.26 | Dead | (15) |
| Yamamoto S | 2006 | 77 | None | Lungs, liver, spleen, heart, adrenal glands,  kidneys, vertebrae, thyroid gland, lymph nodes, pancreas | None | 2 | Dead | (16) |
| Hadgu A | 2010 | 48 | Cough, dyspnea, weight loss | liver, brain | Chemotherapy, Operation | 3 | Dead | (17) |
| Matsukuma S | 2018 | 53 | Cough, chest pain | Lung, brain, stomach | Chemoradiotherapy, operation | 15 | Dead | (18) |
| Matsukuma S | 2018 | 70 | Hemoptysis | Brain, liver, adrenal gland, kidneys | None | 2 | Dead | (18) |
| Matsukuma S | 2018 | 77 | Hemoptysis | Lung, spleen, pancreas | None | 2 | Dead | (18) |
| Matsukuma S | 2018 | 77 | Tumble | None | None | 4 | Dead | (18) |
| Ochi M | 2018 | 60 | Headache, ataxia, dizziness | Brain | Chemotherapy, immunotherapy | 16 | Alive | (19) |
| Nguyen HTT | 2020 | 42 | Chest pain, weight loss | Lung | Operation, chemotherapy (EMA-CO) | 6 | Dead | (20) |
| Iwasaki K | 2022 | 69 | Dyspnea, chest pain, shoulder pain | Lung, liver | None | 3.6 | Dead | (21) |
| Devos B | 2024 | 65 | Cough, gynecomastia, libido loss, agitation | Brain | Chemotherapy, immunotherapy | 9 | Alive | (22) |
| Liang X | 2025 | 67 | Cough, hemoptysis | Lung | Chemotherapy, immunotherapy | 12 | Alive | (23) |
| Takahama M | 2024 | 46 | cough，dyspnea，hemoptysis | diaphragm | Operation | 1 | Dead | (24) |
| Lu H | 2025 | 73 | right limb numbness | liver, brain | Radiotherapy | 3 | Alive | (25) |
| Pan X | 2026 | 60 | cough，expectoration，chest tightness，chest pain | kidney，mediastinum | Chemotherapy, immunotherapy | 6 | Alive | (26) |
| Zeng Y | 2025 | 14 | dyspnea，hemoptysis，chest pain | Lung | Chemotherapy | 16 | Dead | (27) |
| Zeng Y | 2025 | 71 | fatigue and weakness | brain，intestine，heart | Chemotherapy | 15 | Alive | (27) |
|  |  | 41 | Cough | Brain | Chemotherapy ,  radiotherapy,  immunotherapy | 9 | Alive | our case |

**References**

(1)Kamata S, Sakurada A, Sato N, Noda M, Okada Y. A case of primary pulmonary choriocarcinoma successfully treated by surgery. Gen Thorac Cardiovasc Surg (2017) 65(6):361–364. doi:10.1007/s11748-016-0666-8.

(2)Patra SB, Giri DD, Patra BS. Giant choriocarcinoma of the lung. Indian J Chest Dis Allied Sci (1983) 25(3):228–231.

(3)Ma Y, Wang C, Sun PL, Zhu Y, Huang ZK, Jin SX. A case of male primary pulmonary choriocarcinoma. Chin Med J (Engl) (2018) 131(24):3001–3003. doi:10.4103/0366-6999.247205.

(4)Hayakawa K, Takahashi M, Sasaki K, Kawaoi A, Okano T. Primary choriocarcinoma of the lung: case report of two male subjects. Acta Pathol Jpn (1977) 27(1):123–135.

(5)Hattori M, Imura H, Matsukura S, Yoshimoto Y, Sekita K, Tomomatsu T, et al. Multiple-hormone producing lung carcinoma. Cancer (1979) 43(6):2429–2437. doi:10.1002/1097-0142(197906)43:6<2429::AID-CNCR2820430639>3.0.CO;2-I.

(6)Endou T, Ueno H, Okada H, Yano S, Noda M. Primary choriocarcinoma of the lung (a case report of a male subject and review of the literature). Nihon Naika Gakkai Zasshi (1988) 77(9):1404–1409. doi:10.2169/naika.77.1404.

(7)Adachi H, Aki T, Yoshida H, Yumoto T, Wakahara H. Combined choriocarcinoma and adenocarcinoma of the lung. Acta Pathol Jpn (1989) 39(2):147–152. doi:10.1111/j.1440-1827.1989.tb01493.x.

(8)Sridhar KS, Saldana MJ, Thurer RJ, Beattie EJ. Primary choriocarcinoma of the lung: report of a case treated with intensive multimodality therapy and review of the literature. J Surg Oncol (1989) 41(2):93–97. doi:10.1002/jso.2930410208.

(9)Sullivan LG. Primary choriocarcinoma of the lung in a man. Arch Pathol Lab Med (1989) 113(1):82–83.

(10)Durieu I, Berger N, Loire R, Gamondes JP, Guillaud PH, Cordier JF. Contralateral haemorrhagic pulmonary metastases ("choriocarcinoma syndrome") after pneumonectomy for primary pulmonary choriocarcinoma. Thorax (1994) 49(5):523–524. doi:10.1136/thx.49.5.523.

(11)Otsuka T, Ohshima Y, Sunaga Y, Nagashima K. Primary pulmonary choriocarcinoma in a four-month-old boy complicated with precocious puberty. Acta Paediatr Jpn (1994) 36(4):404–407. doi:10.1111/j.1442-200X.1994.tb03210.x.

(12)Toda S, Inoue Y, Ishino T, Yonemitsu N, Terayama K, Miyabara S, et al. A rare case of primary pulmonary choriocarcinoma in a male: immunohistochemical detection for human chorionic gonadotropin, epidermal growth factor and epidermal growth factor receptor. Endocr J (1995) 42(5):655–659. doi:10.1507/endocrj.42.655.

(13)Ikura Y, Inoue T, Tsukuda H, Yamamoto T, Ueda M, Kobayashi Y. Primary choriocarcinoma and human chorionic gonadotrophin-producing giant cell carcinoma of the lung: are they independent entities? Histopathology (2000) 36(1):17–25. doi:10.1046/j.1365-2559.2000.00789.x.

(14)Chen F, Tatsumi A, Numoto S. Combined choriocarcinoma and adenocarcinoma of the lung occurring in a man: case report and review of the literature. Cancer (2001) 91(1):123–129. doi:10.1002/1097-0142(20010101)91:1<123::AID-CNCR16>3.0.CO;2-3.

(15)Tsai JR, Chong IW, Hung JY, Tsai KB. Use of urine pregnancy test for rapid diagnosis of primary pulmonary choriocarcinoma in a man. Chest (2002) 121(3):996–998. doi:10.1378/chest.121.3.996.

(16)Yamamoto S, Tanaka H, Takeo H, Yasuda K, Matsukuma S. Primary pulmonary choriocarcinoma combined with adenocarcinoma. Pathol Int (2006) 56(7):402–407. doi:10.1111/j.1440-1827.2006.01977.x.

(17)Hadgu A, Tindni A, Panda M. Primary pulmonary choriocarcinoma in a male. BMJ Case Rep (2010) 2010:bcr0220102712. doi:10.1136/bcr.02.2010.2712.

(18)Matsukuma S, Obara K, Utsumi Y, Miyai K, Takeo H, Oshika Y, et al. Focal positivity of immunohistochemical markers for pulmonary squamous cell carcinoma in primary pulmonary choriocarcinoma: a histopathological study. Oncol Lett (2018) 16(6):7256–7263. doi:10.3892/ol.2018.9525.

(19)Ochi M, Miyamoto S, Terada Y, Furuhata Y, Awano N, Izumo T, et al. The significant antitumor activity of nivolumab in lung adenocarcinoma with choriocarcinomatous features. Intern Med (2018) 57(12):1773–1777. doi:10.2169/internalmedicine.0002-17.

(20)Nguyen HTT, Hoang HH, Le ATV. A case report of primary pulmonary choriocarcinoma in a man: successful combination of surgery and chemotherapy. Case Rep Oncol (2020) 13(2):923–928. doi:10.1159/000508744.

(21)Iwasaki K, Watanabe K, Kimura H, Yano S. Utility of beta-human chorionic gonadotropin in pleural effusions: report of an autopsy case of a male patient with primary pulmonary choriocarcinoma. Clin Case Rep (2022) 10(12):e6663. doi:10.1002/ccr3.6663.

(22)Devos B, Willemse C, Sterckx M, Debruyne J, Stappaerts I, Van den Mooter T, et al. Beta-hCG secretion by a pulmonary choriocarcinoma in a male patient. Case Rep Oncol Med (2024) 2024:8731806. doi:10.1155/2024/8731806.

(23)Liang X, Xu G, Zhang J, Xu L, Dong L, Wang X, Han W, Chen E. Combination chemotherapy with sintilimab for treatment of a male patient with primary pulmonary choriocarcinoma: a case report and literature review. Front Immunol (2025) 16:1523316. doi:10.3389/fimmu.2025.1523316.

(24)Takahama M. A drastic postoperative course after resection of primary pulmonary choriocarcinoma in a male. Respirol Case Rep (2024) 12(7):e01400. doi:10.1002/rcr2.1400.

(25)Lu H, Jiang A, Liu X, Zhang P, Yu J, Chen D. The role of cranial radiotherapy in managing neurological symptoms of brain metastases from pulmonary choriocarcinoma: a case report and literature review. Discov Oncol (2025) 17(1):39. doi:10.1007/s12672-025-04067-w.

(26)Pan X, Pei Y, Chen T, Xu Y, Shi M. Chemotherapy combined with tislelizumab for a male patient with primary pulmonary choriocarcinoma: a case report and literature review. Discov Oncol (2026) 17(1):318. doi:10.1007/s12672-026-04452-z.

(27)Zeng Y, Zhang H, Pan N, Wang X, Qu L, Xu B, Ye X. Clinicopathological features of primary pulmonary choriocarcinoma in males: a report of two cases and literature review. Respir Med Case Rep (2025) 62:102360. doi:10.1016/j.rmcr.2025.102360.

**Supplementary Table 2. Clinical characteristics of male PPC patients with pretreatment β-HCG data**

| First author | Publication year | Age | Initial β-HCG (mIU/mL) | Survival time (months) | Status | Ref |
| --- | --- | --- | --- | --- | --- | --- |
| Sridhar KS | 1989 | 37 | 3182.00 | 15 | Dead | (1) |
| Toda S | 1995 | 69 | 68920.00 | 1.5 | Dead | (2) |
| Carver C C | 1996 | 69 | 241.00 | 6 | Alive | (3) |
| Nguyen HTT | 2020 | 42 | 2134000.00 | 6 | Alive | (4) |
| Durieu I | 1994 | 61 | 137221.00 | 1.5 | Dead | (5) |
| Iwasaki K | 2022 | 69 | 27000.00 | 3.6 | Dead | (6) |
| Zhu R | 2016 | 67 | 3841.00 | 13 | Alive | (7) |
| Kamata S | 2017 | 70 | 15.00 | 24 | Alive | (8) |
| Otsuka T | 1994 | 0.33 | 180.00 | 7 | Alive | (9) |
| Endou T | 1988 | 37 | 320000.00 | 2.5 | Dead | (10) |
| Okur E | 2002 | 46 | 100.00 | 6 | Alive | (11) |
| Ma Y | 2018 | 71 | 407.23 | 3 | Dead | (12) |
| Iso H | 2023 | 72 | 688.00 | 21 | Alive | (13) |
| Devos B | 2024 | 65 | 491.00 | 9 | Alive | (14) |
| Liang X | 2025 | 67 | 5514.00 | 12 | Alive | (15) |
| Lu H | 2025 | 73 | 49848.00 | 3 | Alive | (16) |
| Pan X | 2026 | 60 | 19857.50 | 6 | Alive | (17) |
| Zeng Y | 2025 | 71 | 1382.00 | 15 | Alive | (18) |

**References**

(1)Sridhar KS, Saldana MJ, Thurer RJ, Beattie EJ. Primary choriocarcinoma of the lung: report of a case treated with intensive multimodality therapy and review of the literature. J Surg Oncol (1989) 41(2):93–97. doi:10.1002/jso.2930410208.

(2)Toda S, Inoue Y, Ishino T, Yonemitsu N, Terayama K, Miyabara S, Sugihara H. A rare case of primary pulmonary choriocarcinoma in a male: immunohistochemical detection for human chorionic gonadotropin, epidermal growth factor (EGF) and EGF receptor. Endocr J (1995) 42(5):655–659. doi:10.1507/endocrj.42.655.

(3)Canver CC, Voytovich MC. Resection of an unsuspected primary pulmonary choriocarcinoma. Ann Thorac Surg (1996) 61(4):1249–1251. doi:10.1016/0003-4975(95)01158-7.

(4)Nguyen HTT, Hoang HH, Le ATV. A case report of primary pulmonary choriocarcinoma in a man: successful combination of surgery and chemotherapy. Case Rep Oncol (2020) 13(2):923–928. doi:10.1159/000508744.

(5)Durieu I, Berger N, Loire R, Gamondes JP, Guillaud PH, Cordier JF. Contralateral haemorrhagic pulmonary metastases ("choriocarcinoma syndrome") after pneumonectomy for primary pulmonary choriocarcinoma. Thorax (1994) 49(5):523–524. doi:10.1136/thx.49.5.523.

(6)Iwasaki K, Watanabe K, Kimura H, Yano S. Utility of beta-human chorionic gonadotropin in pleural effusions: report of an autopsy case of a male patient with primary pulmonary choriocarcinoma. Clin Case Rep (2022) 10(12):e6663. doi:10.1002/ccr3.6663.

(7)Zhu R, Jia C, Yan J, Luo Y, Huo Z. Primary pulmonary choriocarcinoma in a male that was successfully diagnosed and treated: a case report and review of the literature. Medicine (Baltimore) (2016) 95(52):e5693. doi:10.1097/MD.0000000000005693.

(8)Kamata S, Sakurada A, Sato N, Noda M, Okada Y. A case of primary pulmonary choriocarcinoma successfully treated by surgery. Gen Thorac Cardiovasc Surg (2017) 65(6):361–364. doi:10.1007/s11748-016-0666-8.

(9)Otsuka T, Ohshima Y, Sunaga Y, Nagashima K. Primary pulmonary choriocarcinoma in a four-month-old boy complicated with precocious puberty. Acta Paediatr Jpn (1994) 36(4):404–407. doi:10.1111/j.1442-200X.1994.tb03210.x.

(10)Endou T, Ueno H, Okada H, Yano S, Noda M. Primary choriocarcinoma of the lung (a case report of a male subject and review of the literature). Nihon Naika Gakkai Zasshi (1988) 77(9):1404–1409. doi:10.2169/naika.77.1404.

(11)Okur E, Halezeroglu S, Somay A, Atasalihi A. Unusual intrathoracic location of a primary germ cell tumour. Eur J Cardiothorac Surg (2002) 22(4):651–653. doi:10.1016/S1010-7940(02)00390-1.

(12)Ma Y, Wang C, Sun PL, Zhu Y, Huang ZK, Jin SX. A case of male primary pulmonary choriocarcinoma. Chin Med J (Engl) (2018) 131(24):3001–3003. doi:10.4103/0366-6999.247205.

(13)Iso H, Hisakane K, Terashi N, Mikami E, Matsuki S, Sonokawa T, et al. A remarkable response to combination chemotherapy with nivolumab and ipilimumab in a patient with primary pulmonary choriocarcinoma: a case report. Transl Cancer Res (2023) 12(8):2212–2218. doi:10.21037/tcr-23-221.

(14)Devos B, Willemse C, Sterckx M, Debruyne J, Stappaerts I, Van den Mooter T, et al. Beta-hCG secretion by a pulmonary choriocarcinoma in a male patient. Case Rep Oncol Med (2024) 2024:8731806. doi:10.1155/2024/8731806.

(15)Liang X, Xu G, Zhang J, Xu L, Dong L, Wang X, Han W, Chen E. Combination chemotherapy with sintilimab for treatment of a male patient with primary pulmonary choriocarcinoma: a case report and literature review. Front Immunol (2025) 16:1523316. doi:10.3389/fimmu.2025.1523316.

(16)Lu H, Jiang A, Liu X, Zhang P, Yu J, Chen D. The role of cranial radiotherapy in managing neurological symptoms of brain metastases from pulmonary choriocarcinoma: a case report and literature review. Discov Oncol (2025) 17(1):39. doi:10.1007/s12672-025-04067-w.

(17)Pan X, Pei Y, Chen T, Xu Y, Shi M. Chemotherapy combined with tislelizumab for a male patient with primary pulmonary choriocarcinoma: a case report and literature review. Discov Oncol (2026) 17(1):318. doi:10.1007/s12672-026-04452-z.

(18)Zeng Y, Zhang H, Pan N, Wang X, Qu L, Xu B, Ye X. Clinicopathological features of primary pulmonary choriocarcinoma in males: a report of two cases and literature review. Respir Med Case Rep (2025) 62:102360. doi:10.1016/j.rmcr.2025.102360.
